# The Effect of Nordic Hamstring Exercise Intervention Volume on Eccentric Strength and Muscle Architecture Adaptations: A Systematic Review and Meta-analyses

**DOI:** 10.1007/s40279-019-01178-7

**Published:** 2019-09-09

**Authors:** Matthew Cuthbert, Nicholas Ripley, John J. McMahon, Martin Evans, G. Gregory Haff, Paul Comfort

**Affiliations:** 1 Human Performance Laboratory, University of Salford, Greater Manchester, UK; 2 The FA Group, St George’s Park, Burton-upon-Trent, Staffordshire UK; 3 Centre for Exercise and Sports Sciences Research (CESSR), School of Exercise and Health Sciences, Edith Cowan University, Joondalup, Australia; 4 Institute for Sport, Physical Activity and Leisure, Carnegie School of Sport, Leeds Beckett University, Leeds, UK

## Abstract

**Background:**

Although performance of the Nordic hamstring exercise (NHE) has been shown to elicit adaptations that may reduce hamstring strain injury (HSI) risk and occurrence, compliance in NHE interventions in professional soccer teams is low despite a high occurrence of HSI in soccer. A possible reason for low compliance is the high dosages prescribed within the recommended interventions. The aim of this review was to investigate the effect of NHE-training volume on eccentric hamstring strength and biceps femoris fascicle length adaptations.

**Methods:**

A literature search was conducted using the SPORTDiscus, Ovid, and PubMed databases. A total of 293 studies were identified prior to application of the following inclusion criteria: (1) a minimum of 4 weeks of NHE training was completed; (2) mean ± standard deviation (SD) pre- and post-intervention were provided for the measured variables to allow for secondary analysis; and (3) biceps femoris muscle architecture was measured, which resulted in 13 studies identified for further analysis. The TESTEX criteria were used to assess the quality of studies with risk of bias assessment assessed using a fail-safe *N* (Rosenthal method). Consistency of studies was analysed using *I*^2^ as a test of heterogeneity and secondary analysis of studies included Hedges’ *g* effect sizes for strength and muscle architecture variables to provide comparison within studies, between-study differences were estimated using a random-effects model.

**Results:**

A range of scores (3–11 out of 15) from the TESTEX criteria were reported, showing variation in study quality. A ‘low risk of bias’ was observed in the randomized controlled trials included, with no study bias shown for both strength or architecture (*N* = 250 and 663, respectively; *p *< 0.001). Study consistency was moderate to high for strength (*I*^2^ = 62.49%) and muscle architecture (*I*^2^ = 88.03%). Within-study differences showed that following interventions of ≥ 6 weeks, very large positive effect sizes were seen in eccentric strength following both high volume (*g *= 2.12) and low volume (*g *= 2.28) NHE interventions. Similar results were reported for changes in fascicle length (*g *≥ 2.58) and a large-to-very large positive reduction in pennation angle (*g *≥ 1.31). Between-study differences were estimated to be at a magnitude of 0.374 (*p *= 0.009) for strength and 0.793 (*p *< 0.001) for architecture.

**Conclusions:**

Reducing NHE volume prescription does not negatively affect adaptations in eccentric strength and muscle architecture when compared with high dose interventions. These findings suggest that lower volumes of NHE may be more appropriate for athletes, with an aim to increase intervention compliance, potentially reducing the risk of HSI.

## Key Points

**Table:** 

Hamstring strain injury (HSI) accounts for a large proportion of non-contact injuries in team sports including soccer, Australian Rules Football, rugby union, and American Football.
The Nordic hamstring exercise (NHE) has been shown to provide positive architectural and strength adaptations in the hamstrings.
Interventions utilising the NHE have been poorly adopted, particularly by professional soccer teams; potentially due to the high volume prescribed in early interventions.
Despite many interventions prescribing high volumes of the NHE, a larger magnitude of change has been reported using lower, more consistent volumes.

## Background

The investigation of ‘hamstring strain injury’ (HSI) within the scientific literature has been substantial over the last two decades, due to evidence highlighting high HSI occurrence, especially in field-based team sports [1–5]. HSIs accounted for 12% of all injuries reported by 17 top flight European soccer teams [2], 13% of American Football injuries over a 10-year period [3], and 16% of rugby union injuries [5]. Two Australian Football (AF) clubs have also reported 30% of players during one season reported some level of posterior thigh pain [4, 6]. The financial cost of an HSI has been reported to be approximately €250,000 in top-level European soccer clubs for a player that spends 2 weeks out of competition. The cost does not just come from the rehabilitation and salaries of these injured players, but also with lack of availability potentially costing teams with key players not eligible for selection due to injury. Ekstrand et al. [2] indicated that a squad of 25 players would incur approximately seven HSIs in a season. Consequently, further costs could occur with reduced depth of squad becoming an issue or increased injury risk due to players with a low chronic workload suddenly being called upon to play considerable match minutes. As Gabbett [7] outlines, injury risk increases 2–4 times when acute training load is ≥ 1.5 times the chronic workload. Relatively stronger athletes show a reduced risk of injury overall, but possess the ability to tolerate larger changes in load week to week [8]. This increased risk of injury, however, can affect teams around times of fixture congestion with Woods et al. [9] demonstrating that the highest rate of in-season HSI occurs between November and January, traditionally a busy period in English soccer. Teams are also affected towards the end of the season, when training status may have reduced as the importance of results increases. Petersen et al. [10] report injury occurrence to be highest at this period of the season (April–May) in the Danish leagues, where in contrast to English soccer, a winter break is taken. A break in the competitive season, therefore, appears to delay high occurrences of HSI rather than aid in their prevention. The benefits of reducing the fixtures midway through a season are clear, as it reduces the exposure to repeated high intensities during a period of fixture congestion, seen in English soccer leagues over this time, whilst also providing a period in which strength losses that may have occurred due to a greater focus on competition can be reduced.

High-speed running (HSR) activities are reported to be a common cause of HSIs, and it has been revealed that a rate of 60% of HSI reported in professional English soccer were caused by HSR across two seasons [9]. This trend is also observed in English and Australian rugby union, where 68% and 80% of HSI occurrence, respectively, were also caused by this activity [11, 12]. This pattern has also been observed in a single AF team across four seasons when a total of 26 players sustained an HSI due to HSR [6] which is over half of the average 44 player squad. These values may differ dependent upon the threshold at which HSR is determined as anthropometric differences based upon position, particularly in rugby union, may determine a lower or higher maximum speed which means some HSR running may not have been registered due to relative differences. The mechanism behind HSI occurrence during HSR tasks is a failure of the tissues to tolerate the forces applied or required during the task. The primary cause of this intolerance, however, is yet to be determined, with some researchers suggesting a “weak link” approach, whereby an active lengthening (eccentric muscle action) of the sarcomeres creates a chronic accumulative cytoskeletal damage effect until the HSI occurs, whereas other research suggests a more ‘catastrophic’ type event in which the strain occurs due to excessive force applied to the hamstring. There is also a disagreement in the literature as to whether the muscle action involved in the hamstrings during HSR is the active lengthening or eccentric action [13–19], as described above, or whether it is a quasi-isometric action [14, 18, 20]. Despite this lack of clarity, a number of non-modifiable and modifiable risk factors have been outlined [21–23] including, age, ethnicity, previous HSI, fatigue, flexibility, muscle architecture, and strength.

Hamstring strength has been shown to play a major role in increasing or decreasing the risk of HSI [12, 22, 24]. One method of both training and assessing hamstring strength is through the Nordic hamstring exercise (NHE). The NHE is understood to be an eccentric exercise that is performed on the knees with ankles held/strapped with subjects lowering their upper body towards a prone position, as slowly as possible. Opar et al. [25] reported that AF players who produce relative eccentric strength of < 3.45 N kg^−1^ or absolute eccentric strength are < 279 N during the NHE of 4.3–5% more likely to experience an HSI, although this risk decreases by 6.3%, however, for every 10 N increase in force during early pre-season, and by 8.9% by late pre-season. A similar trend was also seen in soccer, with a relative eccentric strength of < 4.35 N.kg^−1^ and absolute eccentric strength of < 337 N increasing injury risk by up to 4.4%. Differences between the injured group and uninjured group were also shown in eccentric hamstring force relative to body mass at the beginning (21% higher in non-injured) and end of their pre-season (17% higher in non-injured).

The force production capabilities of the muscle and the velocity at which this occurs are both influenced by muscle architecture [26]. When described in the literature, muscle architecture involves fascicle length (FL), pennation angle (PA), and either muscle thickness (MT), physiological cross-sectional area (PCSA), or anatomical cross-sectional area (ACSA). The size of the muscle (MT, PCSA, or ASCA) can be influenced by both FL and PA and vice versa depending upon the mode of training. Muscle size typically increases following hypertrophy-based resistance training increasing PA and reduced FL. A decrease in fascicle length, alongside a lack of strength, can increase the risk of injury, as previously mentioned [24]. A review by Opar et al. [23] highlights pertinent architectural aspects of the biceps femoris (BF) explaining that longer fascicles reduce the risk of an injury occurring through overlengthening during eccentric actions [27]; however, the hamstring muscle that lengthens the most during sprinting [28] is the BF long head (BF^LH^), which has shorter fascicles than that of the BF short head (BF^SH^), potentially increasing the susceptibility of the BF^LH^ to injury. It appears that increasing FL has the potential to decrease HSI risk, and in conjunction with eccentric hamstring, strength may reduce this risk.

Eccentric training elicits greater adaptive responses when compared to concentric training regarding both muscle strength and architecture. The differences in adaptations between contraction types are a result of the different mechanisms used to generate force, with eccentric actions occurring due to active lengthening of the fascicles, and concentric actions due to active shortening. The slow eccentric nature of the NHE provides a stimulus, whereby the myosin heads are already attached to actin and forced to detach by the lengthening of the cross bridges, incurring muscle damage [29]. The NHE first appeared in academic literature when Brockett et al. [30] investigated the acute effect of the NHE on angle of peak torque of the hamstrings during eccentric isokinetic assessments. The NHE has since been shown to be an effective injury prevention strategy due to the increase in eccentric strength and subsequent reduction in HSI incidence [23, 31–34]. Many of the published training interventions which have incorporated an NHE protocol have replicated, or used a derivation, of a 10-week protocol outlined by Mjølsnes et al. [33] which resulted in an increase in isometric (7%) and eccentric (11%) isokinetic peak torque and was more beneficial than a concentric comparison (hamstring curl), as there were no changes in hamstring strength within that group. The compliance rate of this study was 96%; however, a subsequent study using the same protocol showed around 60% [35] with other studies not reporting compliance rates. The NHE is maximal in nature and appears to result in a true eccentric mechanism, whereby the hamstring muscles are overloaded past their capacity for maximal eccentric or isometric force production. As a consequence of this maximal eccentric effort, a high level of muscle damage is incurred and subsequent delayed onset muscle soreness (DOMS) is likely to be reported, as eccentric exercise has long been understood to induce muscle soreness [36]. This may influence compliance rates, as Bahr et al. [37] describe a significant minority of teams surveyed for compliance reporting a certain level of resistance from players, as they perceive the exercise to be painful and/or causes fatigue. The intervention outlined by Mjølsnes et al. [33] includes high volumes, with participants performing 700 NHE across 10 weeks, and the combination of high volume and high occurrence of DOMS could be one of the reasons why the same 10-week NHE intervention given to 50 UEFA Champions League clubs has presented a compliance rate as low as 16.7% [37]. Although a minimum dosage has yet to be identified, it is important to compare how various volumes that have been used during interventions affect the hamstrings, with the aim of increasing compliance and ensuring the reduction of HSI. The FIFA 11 + has adopted the NHE in its injury prevention styled warm-up used in soccer, likely due to its practicality as a field-based exercise that requires no equipment; however, this was described as ineffective in some cases due to infrequency of use and lack of motivation from the coaches.

The aim of this systematic review and meta-analyses was to identify NHE-training interventions across all populations, comparing their effectiveness of increasing hamstring strength and altering biceps femoris long head muscle architecture in terms of the volume prescribed throughout the intervention.

## Methods

### Study Design

The design of this systematic review was developed through adhering to the guidelines of the Preferred Reporting Items for Systematic Reviews and Meta-analyses (PRISMA). The PRISMA guideline statement includes a 27-item checklist, designed to be used as a basis for reporting systematic reviews of randomized trials [38]. A review protocol was not pre-registered for this review.

### Literature Search

A Boolean/phrase search mode was utilised using the following keywords: training intervention AND strength AND training AND volume AND eccentric AND hamstrings AND Nordics OR Nordic hamstring exercise OR Nordic curls OR Nordic drops OR Nordic lowers OR Nordic hamstring lowers OR Russian curls. These keywords were applied in the databases PubMed, SPORTDiscus, and Ovid and were filtered to include studies that (1) were presented in peer-reviewed academic journal articles and (2) that were written in the English language. No restrictions were placed upon the age or sex of the subjects, with only an end-date restriction placed upon publication date, due to the NHE being a relatively recent area of research, with the oldest research in this area not yet being surpassed due to any advancements in testing technology.

### Inclusion and Exclusion Criteria

**Fig. 1:**
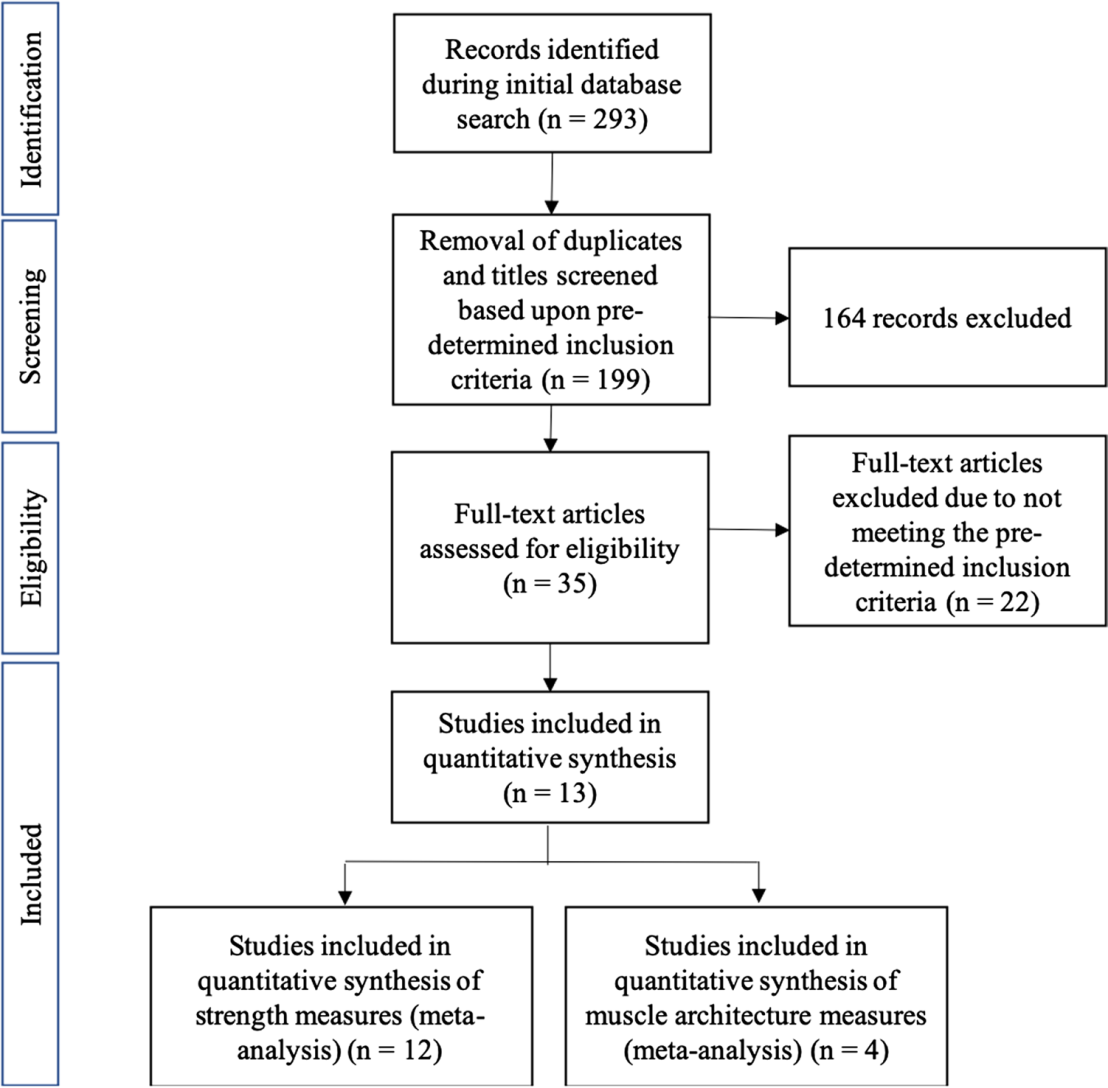
PRISMA flow chart

The primary focus of the literature search was the identification of research studies that implemented an intervention involving the NHE; studies utilising other strength-based protocols in series with the NHE were also accepted. The search timeframe was restricted to 1 December 2018. A total of 293 studies were identified initially for further inspection. Following the removal of duplicate studies, the remaining studies were screened utilising the subsequent criteria. Research was eligible and included within this review, providing that (1) a minimum of 4 weeks of NHE training was completed, (2) mean ± standard deviation (SD) pre- and post-intervention were provided for the measured variables to allow for secondary analysis, and (3) muscle architecture was measured on the BF. Research was excluded due to data being collected through injury incidence questionnaires and not quantifying physiological or performance adaptations. Isokinetic data were also excluded at angular velocities > 120° s^−1^, because reliability and the percentage range of motion at ‘constant velocity’ reduce, as angular velocities increase [39, 40]. A summary of the selection process is outlined in Fig. 1.

### Quality and Risk of Bias Assessment

A subsequent assessment of study quality using the TESTEX scale [41] was then performed by the lead author. The TESTEX scale is an exercise specific scale that has been designed specifically for exercise specialists to assess the quality of reporting of exercise training studies. Two risks of bias assessments were performed through both a fail-safe *N* using the Rosenthal method, and for the randomized controlled trials included within this review, a Cochrane risk of bias assessment tool was used. A Cochrane risk of bias assessment assesses randomized controlled trials based on several categories that include: sequence generation, allocation concealment, blinding of participants and personnel, blinding of outcome assessment, incomplete outcome data, selective outcome reporting, and ‘other issues’; these categories will be graded as ‘high risk of bias’, ‘low risk of bias’, and ‘unclear risk of bias’. A fail-safe number of effects was utilised to calculate the number of un-retrieved null effects that would be needed to diminish the significant of the observed effect at an alpha level of *p *> 0.05. These analyses were conducted using jamovi [42].

### Analysis and Interpretation of Results

Means and standard deviations of strength and architecture measures as well as the duration of the interventions and total prescribed volumes were independently extracted from the included studies. Strength assessment measures included isokinetic variables, including relative eccentric peak torque (at 15° s^−1^, 30° s^−1^, 60° s^−1^, 120° s^−1^), eccentric peak torque (at 60° s^−1^), and eccentric force (in Newtons). Architectural measures included fascicle length, pennation angle, muscle thickness, and muscle cross-sectional area. Effect sizes (ES) were calculated, as they represent standardized values, whereby the magnitude of differences in means between groups or experimental condition can be determined and comparisons made [43]. Hedges’ *g* and the associated 95% CI were used to assess the magnitude of mean differences between pre- and post-interventions, as this accounts for the differences in sample size. Calculation of Hedges’ *g* as an ES was completed using the following formula [44]:

$$g = \frac{{\left( {{\text{Mean}}_{\text{post}} - {\text{Mean}}_{\text{pre}} } \right)}}{{{\text{SD}}_{\text{pooled}} }}.$$

The scale proposed by Hopkins [45] was used for interpretation of the subsequent results, whereby the magnitude of ES was considered as trivial (≤ 0.20), small (0.20–0.59), moderate (0.60–1.19), large (1.20–1.99), or very large (≥ 2.00). Consistency of effects was quantified using a test for heterogeneity (*I*^2^*)* outlined by Higgins et al. [46], whereby a scale of low (< 25%), moderate (25–75%) and high (≥ 75%) *I*^*2*^ values were used for the interpretation of consistency. Estimations for between-study variance were calculated for both strength and architecture using random-effects models for all strength and architecture variables with 95% confidence intervals (95% CI).

## Results

### Search Results

Two hundred and ninety-three titles were identified through databases and reference searches highlighted in the methods section above, and the process that was used to identify the articles reviewed within this systematic review can be seen in Fig. 1 with 94 articles excluded due to duplication, and then, a further 146 titles that did not fit within the predetermined inclusion criteria (see Sect. 2.3) were excluded. Following this the abstracts and full texts were examined against the inclusion criteria, whereby a further 22 articles were excluded leaving the 13 studies included within this review. Data were extracted from the interventions within the included studies from comparisons pre- and post-interventions. Data from testing groups and control groups were extracted; however, this was not a requirement within the inclusion/exclusion criteria, meaning that two of the included studies (i.e., Mjølsnes et al. [33] and Freeman et al. [47]) are without controls.

### Study Quality and Bias Results

**Fig. 2:**
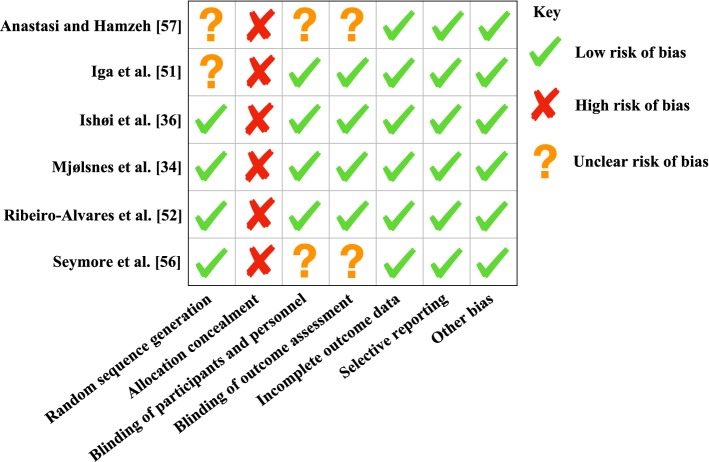
Depiction of the Cochrane risk of bias assessment

Consistency between the studies assessed for both hamstring strength measures and muscle architecture was moderate to high, with *I*^2^ values of 62.49% and 88.03%, respectively. Quality of assessment was assessed using the TESTEX criterion (see Table 1); the mean score for the studies in this review was six out of a total 15, with the highest scoring study being a randomized controlled trial [35] with a score of 11. Two risk of bias assessments were also performed, the first (Cochrane risk of bias assessment tool) showing a low risk of bias overall within the randomized controlled studies included in this review (Fig. 2), the second identifying the results of this meta-analysis are not subject to publication bias (*p *< 0.001) with 250 and 663 “filed-away” studies needed to prove null effects of NHE interventions on strength and architecture, respectively.

### Systematic Review and Meta-analyses Findings

**Fig.3:**
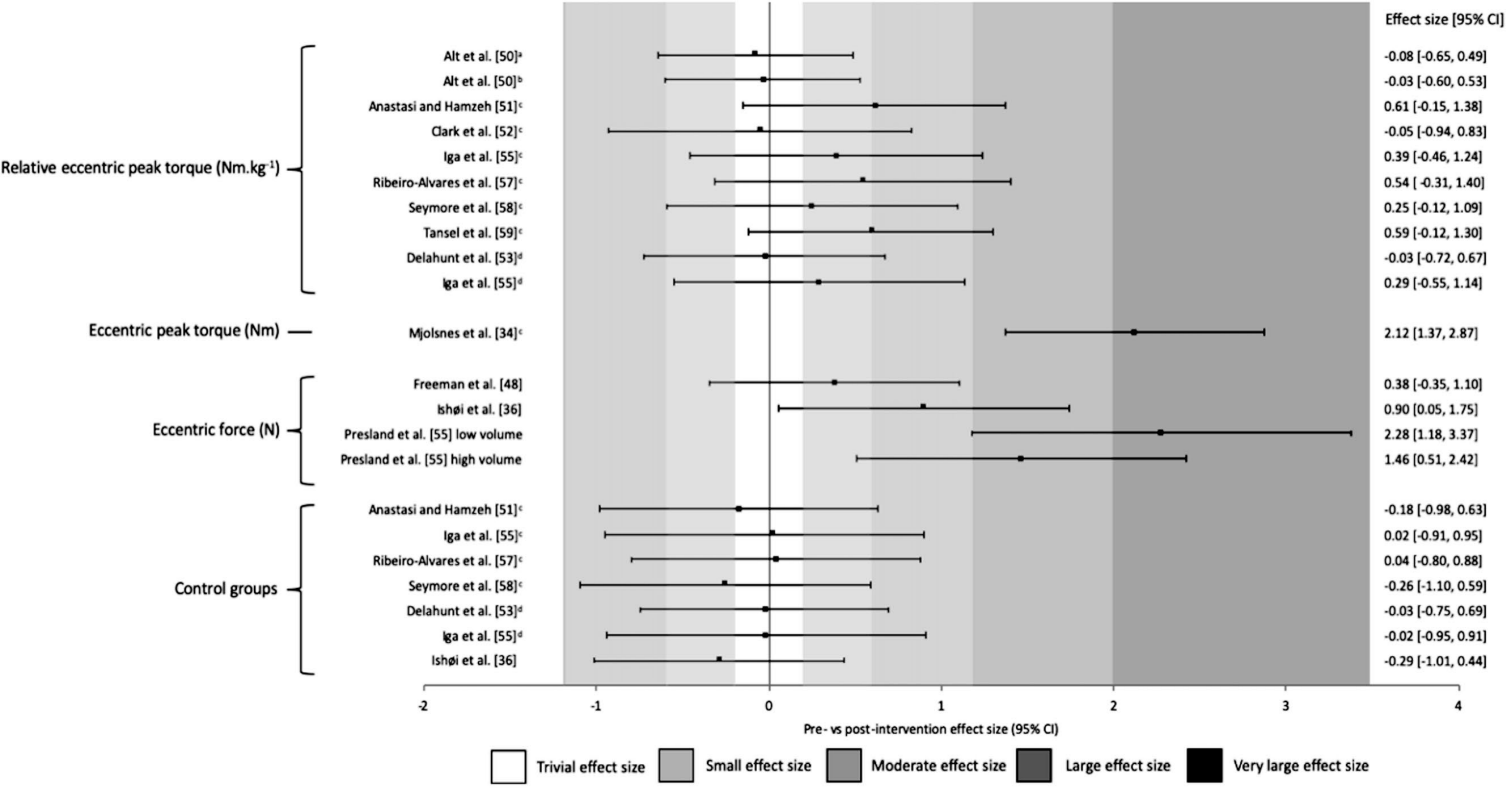
Changes in strength pre- and post-Nordic hamstring exercise intervention. *Nm kg*^*−1*^ Newton meters per kilogram, *Nm* Newton meters, *N* Newtons, *CI* confidence interval, ^a^15° s^−1^, ^b^30° s^−1^, ^c^60° s^−1^, ^d^120° s^−1^

Within-study differences pre–post-intervention is shown in the figures below. Figure 3 illustrates the magnitude of the change in strength (ES and 95% CI) of all eight eccentric hamstring strength variables collected across thirteen studies. Only one study which assessed eccentric peak torque showed very large increases (*g *= 2.12) post-intervention, with two studies that assessed isometric peak torque resulting in large increase post-intervention (*g *= 1.80 and 1.29). In contrast, large to very large (*g* ≥ 1.32) increases in eccentric force during the NHE were evident post-intervention in the two studies that used this metric. Control groups in all studies showed trivial or negative changes in torque or force (*g *= ≤ 0.14). The pooled summary of variance from the random-effects model was 0.374 (*p *= 0.009, 95% CI 0.94–0.655) for strength and 0.793 (*p *< 0.001, 95% CI 0.338–1.248) for muscle architecture.

**Fig. 4:**
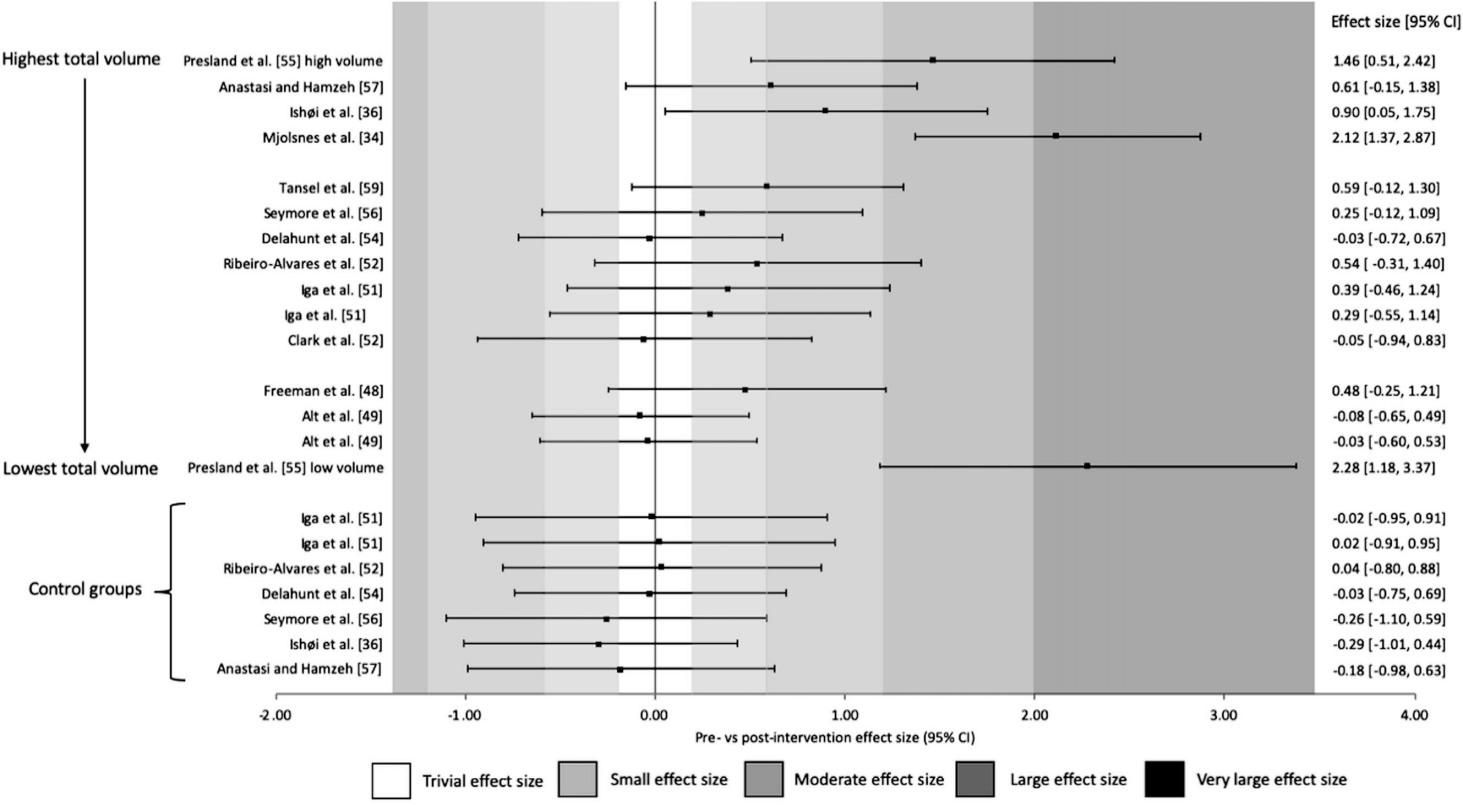
Changes in strength pre- and post-Nordic hamstring exercise intervention ranked from highest to lowest total volumes. *CI* confidence interval

**Fig. 5:**
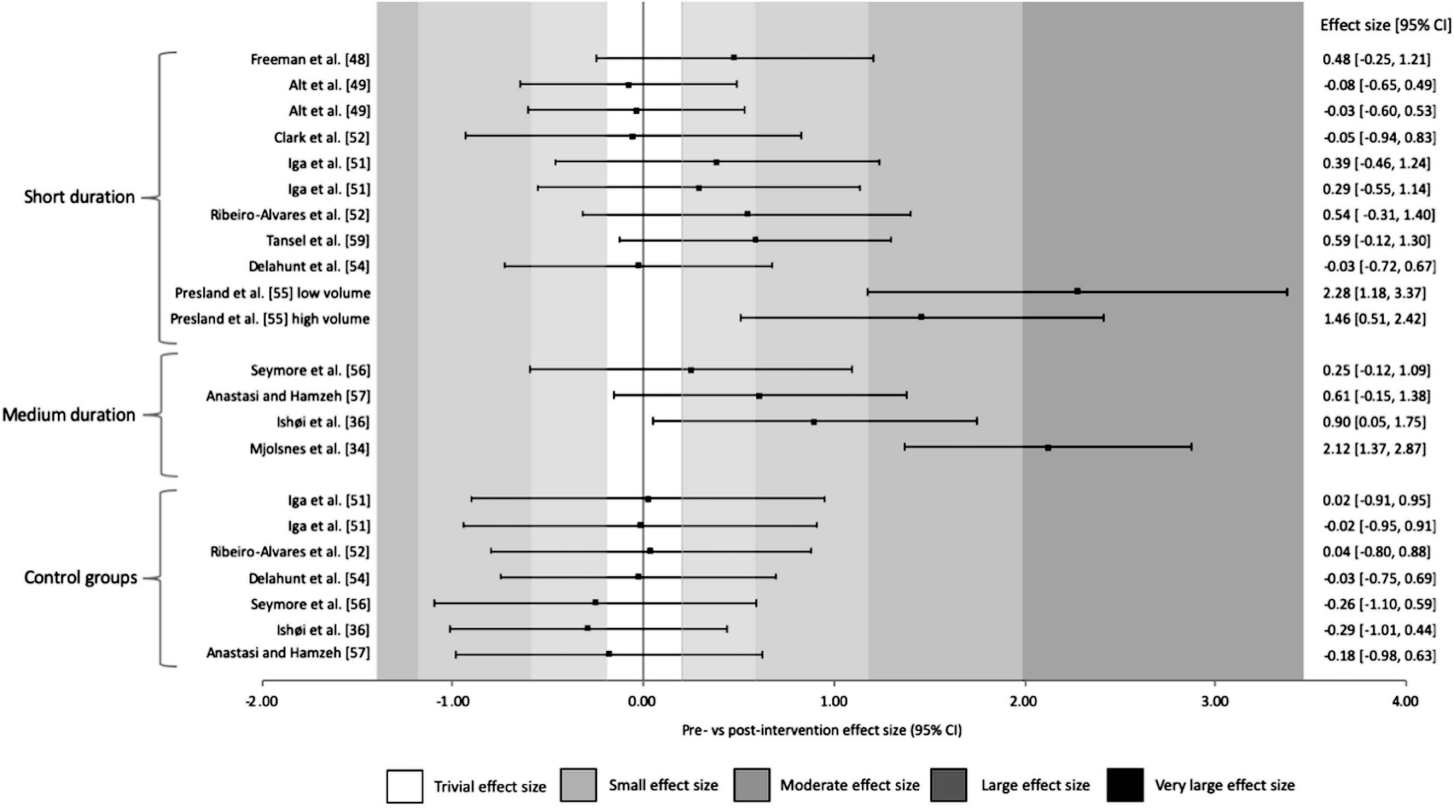
Changes in strength pre- and post-Nordic hamstring exercise intervention ranked from short duration (4–6 weeks) to medium duration (8–10 weeks). *CI* confidence interval

Figures 4 and 5 illustrate the same results as shown in Fig. 3; however, these are ordered in terms of volume (highest to lowest) prescribed during the interventions (Fig. 4) and duration (shortest to longest) of the interventions (Fig. 5). No trend with respect to volume was revealed, with both high and low volumes resulting in very large ES; however, a threshold of 6 weeks as a minimum intervention duration was detected. Out of the eight studies [47–54] in the short duration group (4–6 weeks), those that only prescribed a 4-week intervention found trivial to small differences in pre-to-post-intervention. Large-to-very large ES were seen only in the single 6-week study [54] and moderate to very large increases in the medium duration studies (8–10 weeks) [33, 35, 55, 56].

**Fig. 6:**
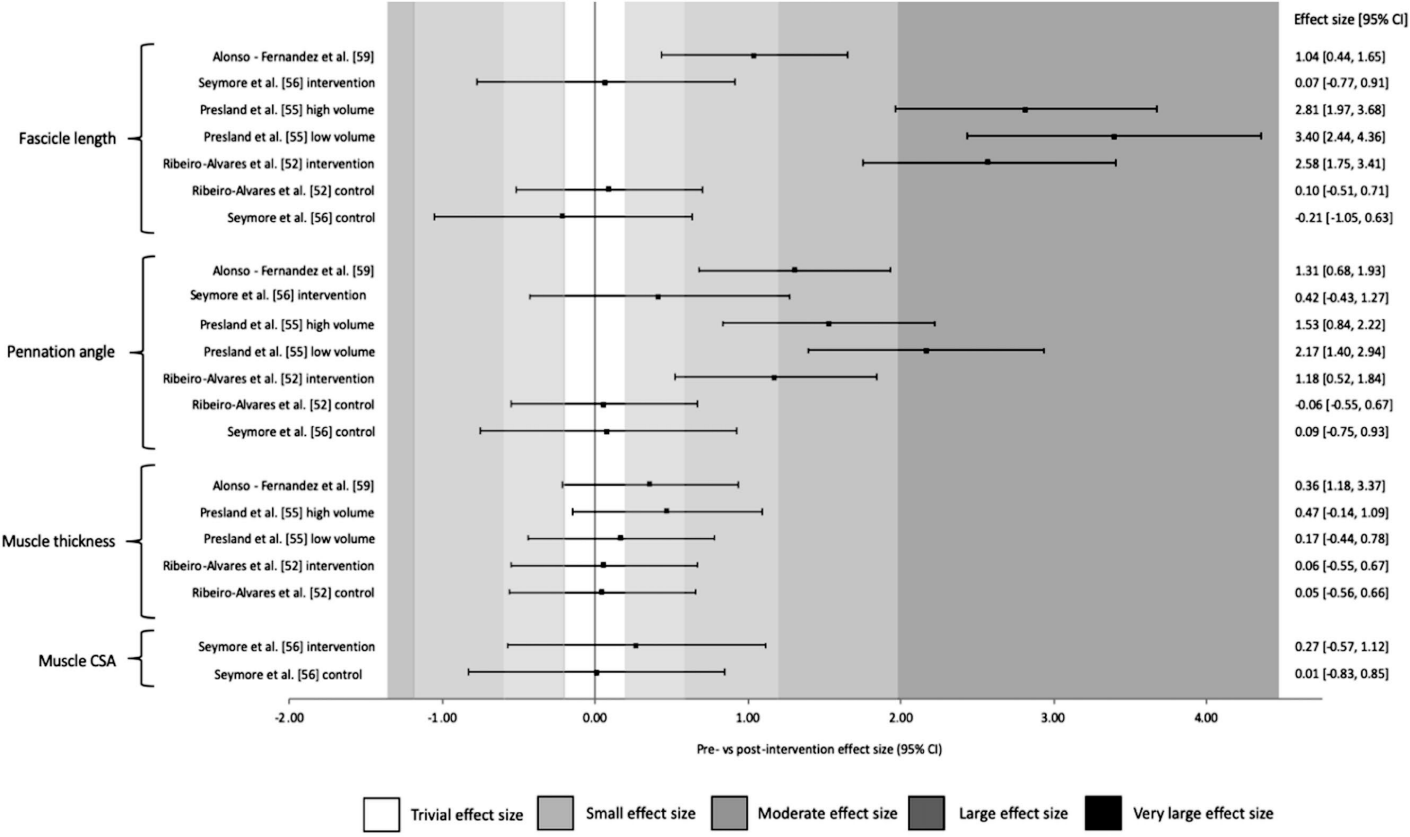
Changes in muscle architecture pre- and post-Nordic hamstring exercise intervention. *CSA* cross-sectional area, *CI* confidence interval

Muscle architecture ES is demonstrated in Fig. 6, with 3 measures of FL showing very large ES (*g* ≥ 2.58). Positive improvements (a reduction) in PA can be seen with large-to-very large ES (*g* ≥ 1.31), with no meaningful change (*g* ≤ 0.47) in muscle size (MT or PCSA/ACSA). Similar to the strength variables, the control groups also showed either trivial or small negative ES.

## Discussion

The purpose of this review and meta-analysis was to identify the effect of training volume, prescribed through an NHE intervention, on changes in eccentric hamstring strength and BF^LH^ muscle architecture. The evidence collected suggests that both high and low volume prescription can produce large-to-very large improvements in both strength (eccentric torque and eccentric force) and muscle architecture (FL and PA) over a minimum duration of 6 weeks. The quality and bias of the studies reviewed, assessed using the TESTEX scale, showed a large variation with many of the studies scoring less than half of the total available. The majority of studies failed to report levels of compliance, activity levels of control groups, and did not use a blind assessor or report concealment of group allocation (Table 1).

**Table 1:** Study scores allocated based on TESTEX criteria [41]

Study	1 (1 point)	2 (1 point)	3 (1 point)	4 (1 point)	5 (1 point)	6 (3 points)	7 (1 point)	8 (2 points)	9 (1 point)	10 (1 point)	11 (1 point)	12 (1 point)	Total
TESTEX criterion													
Alonso-Fernandez et al. [58]	1	0	0	0	0	0	0	0	1	0	1	1	4
Alt et al. [58]	1	0	0	0	0	0	0	2	1	0	1	1	6
Anastasl and Hamzeh [56]	1	0	0	1	0	0	0	2	1	0	1	1	7
Clark et al. [51]	1	0	0	0	0	0	0	0	1	0	1	1	4
Delahunt et al. [53]	1	1	0	1	0	0	0	0	0	0	1	1	5
Freeman et al. [47]	0	0	0	1	0	1	0	1	1	0	1	1	6
lga et al. [50]	0	0	0	1	0	0	0	2	1	0	1	1	6
Ishøi et al. [35]	1	1	1	1	1	1	0	2	1	0	1	1	11
Mjolsnes et al. [33]	0	0	0	0	0	2	0	2	1	0	1	1	7
Presland et al. [54]	1	0	0	1	0	2	0	2	1	0	1	1	9
Ribeiro-Alvares et al. [51]	1	1	0	1	0	0	0	2	1	0	1	1	8
Seymore et al. [55]	1	0	0	1	0	0	0	2	1	0	1	1	7
Tansel et al. [58]	0	0	0	0	0	0	0	0	1	0	1	1	3

*1* eligibility criteria specified, *2* randomization specified, *3* allocation concealment, *4* groups similar at baseline, *5* blinding of assessor; *6* outcome measures assessed in 85% of subjects, *7* intention-to-treat analysis, *8* between group statistical comparisons reported, *9* point measures and measures of variability of outcomes reported, *10* activity monitoring in control groups reported, *11* relative exercise intensity retained constant, *12* exercise volume and energy expenditure

### The Effect of Volume on Eccentric Strength

A wide range of volumes and durations were identified following the literature search (see Table 1 for a summary). An average weekly volume across interventions ranged from 21 repetitions a week to as high as 73 repetitions a week. The majority of studies within this review, however, built up the volume throughout with highest weekly volumes reaching 90–100 repetitions. Further investigation showed that repetitions within sets reached between 8 and 12 towards the end of many of the interventions. The risk of incurring an HSI is increased due to a lack of hamstring strength; however, traditional strength training would recommend ≤ 6 repetitions at ≥ 85% of one repetition maximum (RM) according to National Strength and Conditioning Association (NSCA) guidelines [57] with an increase in intensity producing the most effective increases in force production capabilities. The NHE being supramaximal or conceptually ‘above 1RM’, the assumption would be that fewer repetitions would need to be produced to create the same stimulus.

The two studies resulting in the greatest increase in eccentric hamstring strength [33, 54] differed in the prescribed volume with high volumes applied during the intervention by Mjølsnes et al. [33] and the low volumes by Presland et al. [54]; however, both included periods of ≥ 4 weeks, whereby volume was not increased, unlike the other interventions identified in this systematic review. With no increase in volume (sets and/or repetitions), the intensity of the exercise may have increased as a result of an increasing breakpoint angle (the angle at which the hamstrings inhibit, and the upper body falls to the floor), i.e., the individual gets closer to the ground before falling which in turn increases the torque due to force being applied over a greater moment. This increase in intensity, much with traditional strength training, is likely to be the reason for the effectiveness of these two programmes. Understanding intensity may be an issue when prescribing NHE volume because of the inverse relationship, it has with rate of perceived exertion (RPE). Although RPE may be relatively high initially when performing the NHE, it is likely to increase with an increase in repetitions. Conversely, although intensity should always be supramaximal, force production is likely to decrease due to fatigue and a reduction in the breakpoint angle, meaning that the amount of “work done” may reduce. Even with high levels of fatigue, athletes can still perform the movement to an extent, resulting in the perception of high levels of exertion. Ishøi et al. [35] replicated the intervention designed by Mjølsnes et al. [33], but observed only moderate improvements in eccentric strength across the 10 weeks; this, however, was likely due to the compliance of the subjects within the studies with Ishøi et al. [35] reporting only 60% compliance compared to the 96% reported by Mjølsnes et al. [33].

Potential conflict in the findings may be related to methodological differences when testing eccentric strength, combinations of eccentric force, and relative peak torque at various angles and angular velocities are reported. Poor agreement (*r *= 0.35) between eccentric force collected during the NHE and isokinetic eccentric peak torque at 60° s^−1^ has been reported previously [59], suggesting a difference between the two methods of assessing eccentric force production. Although there were very large improvements both in terms of isokinetic torque and eccentric force, the greatest improvements across studies were seen in eccentric force, which was due to the testing procedure being an NHE, whereby a learning effect in addition to any improvements in strength may have been present. When considering the discrepancy between angular velocities tested, this may have had an effect when considering its applicability to both the exercise and sprinting as a task associated with HSI; however, given that this analysis only evaluated effect sizes pre- and post-intervention and the testing procedures being consistent in all studies this is unlikely to have had any influence on the findings of this review.

### The Effect of Volume on Muscle Architecture

Despite high training volumes being associated with hypertrophic responses, no interventions identified had any meaningful effect on either MT or muscle CSA, which could have been a result of relatively short intervention durations with the longest being 8 weeks (see Table 2). In contrast, there were very large increases in FL attributed to three different volumes, including both the highest based on the average weekly volume and the lowest [54] between pre and post-intervention. This was also true of PA, which was associated with large to very large decreases appearing as a positive ES in Fig. 6, due to a decrease in PA being the desired outcome. Presland et al. [54] showed no statistical differences (*P *= 0.982) in the increases in FL between the high and low volume groups; however, the lower volume group showed significantly greater (*P *< 0.001) decreases in PA pre–post-intervention compared to the changes reported in the higher volume group.

**Table 2:** Characteristics of the Nordic hamstring interventions used in studies included in this review

Study	Subjects	Population	Duration (weeks)	Intervention (reps × sets × frequency)	Total volume (reps)	Average weekly volume (reps)	Outcomes
Alonso-Fernandez et al. [58]	*n* = 23	Recreationally active males	8	Week 1–2 = 2 × 6 × 2 Week 3–4 = 3 × 4–6 × 3 Week 5–6 = 3 × 8 × 3 Week 7–8 = 3 × 10 × 3	480	60	Significant increases in FL and MT of the BFlh and significant decreases in PA
Alt et al. [48]	*n* = 16	Regional to national level male sprinters	4	Week 1–4 = 3 × 3 × 3	108	27	No significant increases in peak torque as a result of the intervention
Anastasi and Hamzeh [56]	*n* = 24	Amateur female rugby union players	10	Week 1–2 = 3 × 6 × 3 Week 3–4 = 3 × 7 × 3 Week 5–7 = 3 × 8 × 3 Week 8–10 = 3 × 10 × 3	720	72	Increases in peak torque of both limbs pre to post
Clark et al. [51]	*n* = 9	Amateur Australian Rules Football players	4	Week 1 = 2 × 5 × 1 Week 2 = 2 × 6 × 2 Week 3 = 3 × 6 × 3 Week 4 = 3 × 8 × 3	160	40	Significant reductions in peak torque pre to post
Delahunt et al. [53]	*n* = 29	Recreationally active males	6	Week 1 = 2 × 5 × 1 Week 2 = 2 × 6 × 2 Week 3 = 3 × 6 × 3 Week 4 = 3 × 8 × 3 Week 5–6 = 3 × 12, 10, 8 × 3	340	57	Significant increases in peak torque pre to post, with large effect sizes
Freeman et al. [47]	*n* = 28	Team sport adolescent athletes	4	Week 1 = 2 × 5 × 2 Week 2 = 3 × 4 × 2 Week 3 = 3 × 5 × 2 Week 4 = 3 × 6 × 2	110	28	Significant increases in peak eccentric force pre to post, with small effect sizes
Iga et al. [50]	*n* = 18	English professional male soccer players	4	Week 1 = 2 × 5 × 1 Week 2 = 2 × 6 × 2 Week 3 = 3 × 6 × 3 Week 4 = 3 × 8 × 3	160	40	Significant increases in peak torque at 3 different angular velocities pre to post
Ishøi et al. [35]	*n* = 35	Amateur male soccer players	10	Week 1 = 2 × 5 × 1 Week 2 = 2 × 6 × 2 Week 3 = 3 × 6–8 × 3 Week 4 = 3 × 8–1 × × 3 Week 5–10 = 3 × 12, 10, 8 × 3	700	70	Significant increases in peak eccentric hamstring force pre to post
Mjølsnes et al. [33]	*n* = 21	Competitive male soccer players	10	Week 1 = 2 × 5 × 1 Week 2 = 2 × 6 × 2 Week 3 = 3 × 6–8 × 3 Week 4 = 3 × 8–10 × 3 Week 5–10 = 3 × 12, 10, 8 × 3	700	70	Significant increases in peak torque pre to post. Significantly greater increases compared to concentric exercise
Presland et al. [54] (high volume)	*n* = 20	Recreationally active males	6	Baseline week 1–2 = 4 × 6 × 2 Week 3 = 4 × 8 × 2 Week 4 = 4 × 10 × 2 Week 5–6 = 5 × 10 × 2	392	73	Significant increases in BFFL for both groups, and decreases in PA in the pre to post for the low volume group. No significant differences were observed for MT. Eccentric hamstring force also increased significantly in both groups
Presland et al. [54] (low volume)				Baseline week 1–2 = 4 × 6 × 2 Week 3–6 = 2 × 4 × 1	80	21	
Ribeiro-Alvares et al. [51]	*n* = 20	Healthy young adults (aged 18–35)	4	Week 1 = 3 × 6 × 2 Week 2 = 3 × 7 × 2 Week 3 = 3 × 8 × 2 Week 4 = 3 × 10 × 2	186	47	Significant increases in peak torque pre to post, with moderate effect sizes. BFMT did not change pre to post, FL did increase and PA decrease to a very large and large effect size, respectively
Seymore et al. [55]	*n* = 20	Recreationally active adults	6	Week 1 = 2 × 5 × 1 Week 2 = 2 × 6 × 2 Week 3 = 3 × 6 × 3 Week 4 = 3 × 8 × 3 Week 5–6 = 3 × 12, 10, 8 × 3	340	57	No significant increases in peak torque as a result of the intervention. BF FL and PA both increased without significance, CSA increased significantly
Tansel et al. [58]	*n* = 26	Healthy boys (aged 10–12)	5	Week 1 = 2 × 5 × 1 Week 2 = 2 × 6 × 2 Week 3 = 3 × 6–8 × 3 Week 4 = 3 × 8–10 × 3 Week 5 = 3 × 12, 10, 8 × 3	286	57	Significant increases in hamstring peak torque pre to post

*reps* repetitions; *FL* fascicle length, *MT* muscle thickness, *BF* biceps femoris, *PA* pennation angle, *CSA*  cross-sectional area

Trivial to small changes in ES of FL and PA were only observed by Seymore et al. [55], who consequently claimed that an increase in FL is unlikely to be responsible for the injury protection that an NHE provides. This statement, however, does not consider other factors that may have influenced the lack of change in muscle architecture and eccentric hamstring strength in this study. First, both FL and PA were assessed as an average of the proximal, middle and distal portions of the hamstring, which would not have taken into consideration any regional improvements that other studies may have found by typically assessing just the muscle belly or ‘middle’ portion. Seymore et al. [55] also reduced the data down into a group of ‘responders’ and ‘non-responders’ to outline the individualistic changes in the experimental groups. The group termed ‘responders’ showed considerably lower relative eccentric hamstring torque compared to the non-responder pre-intervention. Subsequently, the responders showed significantly large increases in both CSA and PA (*p* ≤ 0.008 and *d* ≥ 1.34) and a slight reduction in FL, suggesting that a hypertrophic response much like a non-trained athlete would demonstrate, whereas the group termed ‘non-responders’, despite a reduction in strength, did show large increases in FL (*d *= 1.65). This appears to contradict their argument somewhat, as there was an increase in FL, just not in the weaker group, but a subsequent lower volume strength focused programme may provide those changes. Unfortunately, these groups were only a fraction of the sample size, and it is, therefore, recommended that further investigations determine if differential changes occur in strong versus weak subjects.

### Compliance and DOMS

As previously highlighted in Sect. 4.1, compliance in terms of real-world application of NHE interventions or hamstring injury prevention programmes is low [37]. In this systematic review, low compliance was reported by Ishøi et al. [35] who achieved only 60% compliance during their intervention. Not all studies have reported compliance; however, this could be a potential cause for those studies that have demonstrated low effectiveness of interventions and not reported compliance, given that the high levels of effectiveness of other interventions included within this review. A common reason for athletes not complying with NHE interventions or coaches not prescribing the NHE at all is the high levels of DOMS experienced as a consequence of the large eccentric stimulus the exercise provides. Three studies [33, 47, 48] identified DOMS or muscle soreness through a visual analogue scale which is used as a scale for pain, with the data being obtained following each warm-up prior to the intervention sessions. The scores recorded were no higher than six out of ten, attributing the soreness to moderate-to-low levels of pain. Both pain level and the difference between soreness and pain are subjective measures. In the study by Ishøi et al. [35], subjects who reported hamstring pain were removed from the study, which would have been the reason for low compliance. Further clarification needs to be conducted within studies as to whether this is just due to a high level of muscle soreness, rather than pain due to injury, as it may then be necessary to provide a longer period prior to any intervention to allow for a gradual increase in exposure to the exercise.

### Study Quality and Bias

The variability of the quality of studies included in this review, as highlighted by the TESTEX scores provided (Table 1), allows for the identification of potential discrepancies when comparing studies. The common shortfalls throughout the range of studies are based around the methodological control of concealing group allocation and blinding assessors, as well as monitoring activity within the control groups and outside the training intervention. The blinding of assessors may not be viable in terms of the strength measures; however, when considering the muscle architecture measurements, there may be some element of assessor bias, as the measurements are somewhat subjective. Due to the nature of most interventions being applied within a ‘real-world’ type setting, it is important to include any concurrent training stimuli to fully assess if there were any outside interactions with any adaptations seen following an intervention. In the TESTEX criteria, the reporting of compliance and adherence is given a greater portion of the scores; however, only four studies scored at least one point out of a possible three within the section. The low compliance of studies could explain low effectiveness and reporting on compliance may prevent inaccurate conclusions being drawn about the possible impact of such interventions. Publication bias was not present in the studies included within this review, as highlighted in the results, with Fig. 2 also depicting a ‘low risk of bias’ for the majority of the seven categories, the only ‘high risk of bias’ identified being through the allocation concealment. Allocation concealment refers to both participants and investigators enrolling participants not foreseeing group allocation; however, this is not always ecologically valid, especially within a sporting club setting which is consistent across all the included studies. The studies categorized as ‘unclear’ were due to a lack of detail and description within those studies.

## Conclusion

Many NHE interventions are derived from the original model provided by Mjølsnes et al. [33], which is understandable due to the effectiveness shown for increasing both hamstring strength and FL whilst reducing PA. This, however, has not been effectively replicated since, and whether that is attributable to lower compliance throughout the intervention or reducing the length of the intervention, so that it does not allow for a plateau in training volume, and it seems that a new approach is needed in order for the general compliance rate in real-world application to increase. This review has shown that a reduction in overall training volume need not have a negative effect; however, the focus needs to be on allowing the intensity of the exercise to increase, as would occur in traditional strength training. Allowing players to ‘get better’ at the exercise by keeping the training volume consistent, to allow them to slowly produce force over a greater range of motion, seems to be the most effective way of producing the desired adaptations. Although a minimal dosage is yet to be determined, the recommendations based on the evidence found in this review would be to reduce the training volume and keep it at a consistent level if the lack of compliance in players is due to severe DOMS. Although outside the scope of this review, it is important to consider that the NHE should be part of a wider holistic hamstring programme to account for both strength, architectural adaptations, and ability to withstand high velocity actions that are observed within sporting actions. Future studies should investigate how the frequency of NHE prescription effects adaptations whilst also looking to increase study quality.

## Data Availability

The data within this study are secondary data and available through the relevant articles referenced throughout. All statistical analysis was carried out using Jamovi [42] an open source software that is freely available.
