# Corrections to: The Effect of Nordic Hamstring Exercise Intervention Volume on Eccentric Strength and Muscle Architecture Adaptations: A Systematic Review and Meta-analyses

**DOI:** 10.1007/s40279-019-01208-4

**Published:** 2019-11-07

**Authors:** Matthew Cuthbert, Nicholas Ripley, John J. McMahon, Martin Evans, G. Gregory Haff, Paul Comfort

**Affiliations:** 1 Human Performance Laboratory, University of Salford, Greater Manchester, UK; 2 The FA Group, St George’s Park, Burton-upon-Trent, Staffordshire UK; 3 Centre for Exercise and Sports Sciences Research (CESSR), School of Exercise and Health Sciences, Edith Cowan University, Joondalup, Australia; 4 Institute for Sport, Physical Activity and Leisure, Carnegie School of Sport, Leeds Beckett University, Leeds, UK

## Correction to: Sports Medicine 10.1007/s40279-019-01178-7

**Page 5, column 1, section 3.2, paragraph 1, sentence 1:** The following sentence, which previously read:

“Consistency between the studies assessed for both hamstring strength measures and muscle architecture was moderate to high, with *I*^2^ values of 62.49% and 88.03%, respectively.”

Should read:

“Consistency between the studies assessed for both hamstring strength measures and muscle architecture was moderate to high, with *I*^2^ values of 58.58% and 88.03%, respectively.”

**Page 5, columns 1–2, section 3.2, paragraph 1, sentence 3:** The following sentence, which previously read:

“Two risk of bias assessments were also performed, the first (Cochrane risk of bias assessment tool) showing a low risk of bias overall within the randomized controlled studies included in this review (Fig. 2), the second identifying the results of this meta-analysis are not subject to publication bias (*p* < 0.001) with 250 and 663 “filed-away” studies needed to prove null effects of NHE interventions on strength and architecture, respectively.”

Should read:

“Two risk of bias assessments were also performed, the first (Cochrane risk of bias assessment tool) showing a low risk of bias overall within the randomized controlled studies included in this review (Fig. 2), the second identifying the results of this meta-analysis are not subject to publication bias (*p* < 0.001) with 178 and 663 “filed-away” studies needed to prove null effects of NHE interventions on strength and architecture, respectively.”

**Pages 5–6, columns 2 (page 5) and 1 (page 6), section 3.3, paragraph 1, sentence 6:** The following sentence, which previously read:

“The pooled summary of variance from the random-effects model was 0.374 (*p* = 0.009, 95% CI 0.94–0.655) for strength and 0.793 (*p* < 0.001, 95% CI 0.338–1.248) for muscle architecture.”

Should read:

“The pooled summary of variance from the random-effects model was 0.439 (*p* = 0.001, 95% CI 0.160–0.709) for strength and 0.793 (*p* < 0.001, 95% CI 0.338–1.248) for muscle architecture.”

**Page 11, Table 1, Alt et al. [58] row:** The cell entry in column 1, which previously read:

“Alt et al. [58]”

Should read:

“Alt et al. [48]”

